# Supplementary figures and images for: Changes in SARS-CoV-2 viral load and mortality during the initial wave of the pandemic in New York City
Source: PLoS One. 2021 Nov 19;16(11):e0257979. doi: 10.1371/journal.pone.0257979 (PMC8604305; doi:10.1371/journal.pone.0257979)

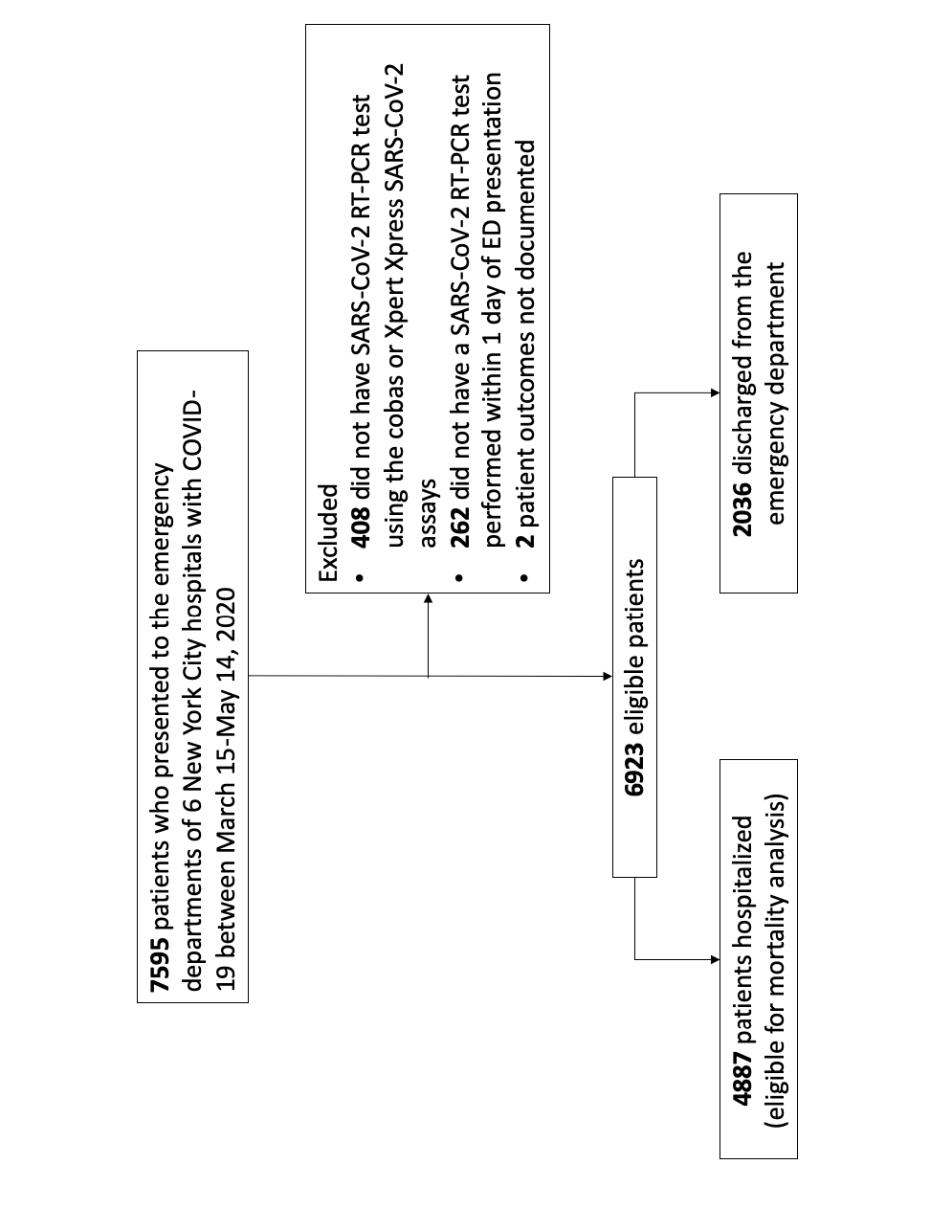

Supplement: S1 Fig — (TIFF) [file pone.0257979.s001.tiff]

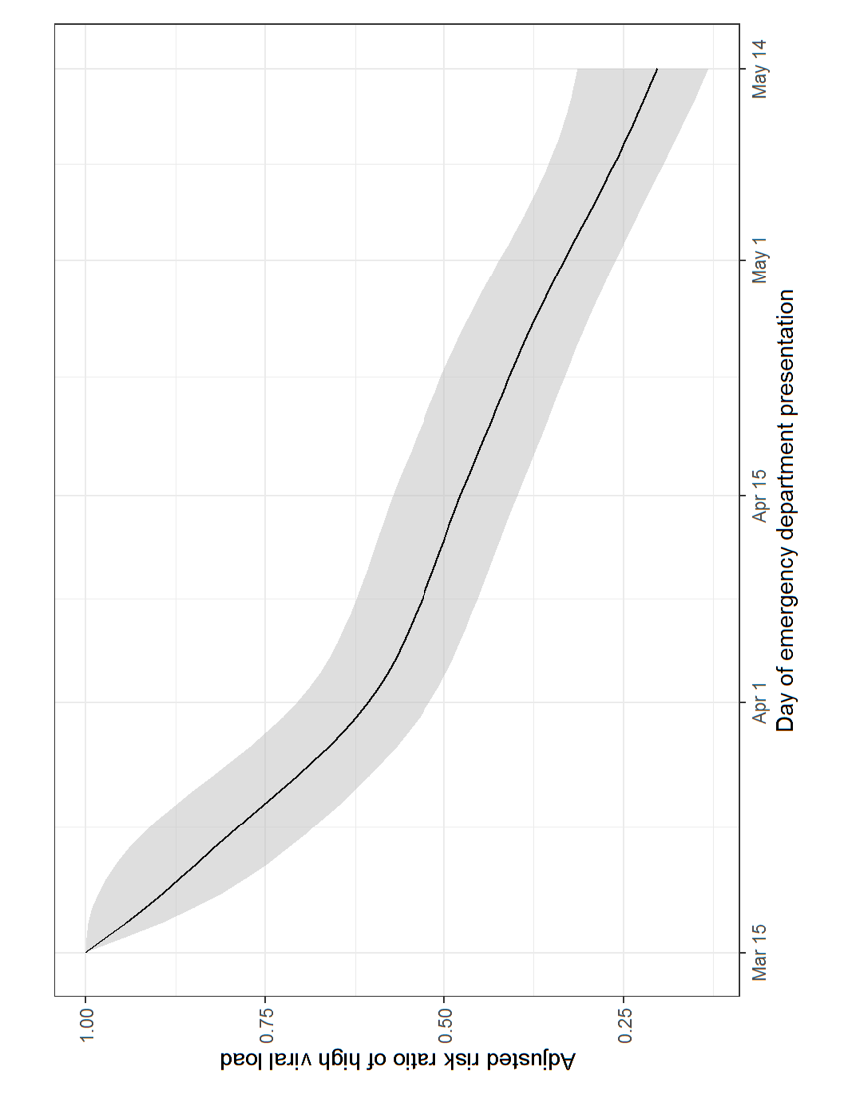

Supplement: S2 Fig — This model adjusted for age, gender, comorbidities, type of RT-PCR assay, and hospital of presentation. In the gamma regression model for CT values, the location of ED presentation (p = 0.93) and the interaction between day of ED presentation and assay type (p = 0.85) were insignificant. (TIFF) [file pone.0257979.s002.tiff]

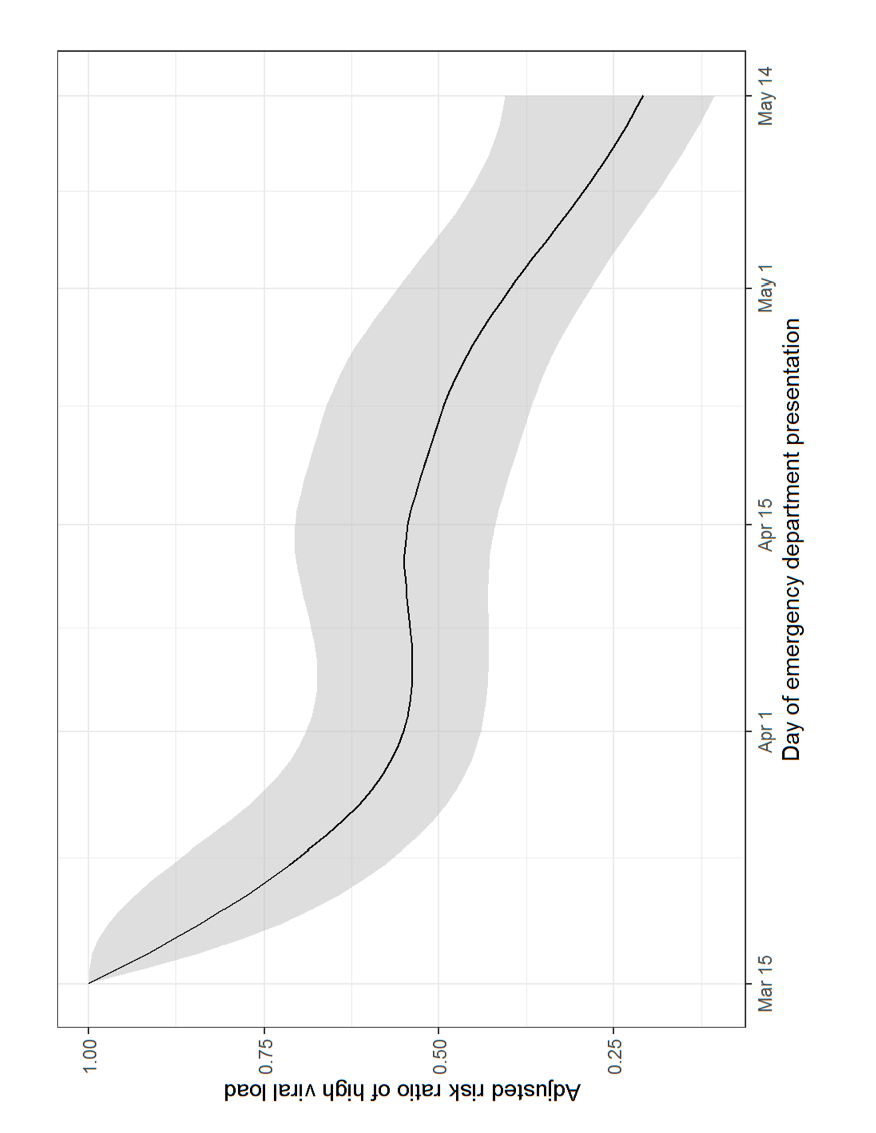

Supplement: S3 Fig — This model adjusted for the variables in S2 Fig plus duration of symptoms, including only the 50.5% of patients for whom duration of symptom data were available. (TIFF) [file pone.0257979.s003.tiff]
