# Supplementary material for: Changes in SARS-CoV-2 viral load and mortality during the initial wave of the pandemic in New York City
Source: PLoS One. 2021 Nov 19;16(11):e0257979. doi: 10.1371/journal.pone.0257979 (PMC8604305; doi:10.1371/journal.pone.0257979)
Supplement: S1 Table — (DOC) [file pone.0257979.s004.doc]

**S1 Table. Characteristics of Patients Presenting with COVID-19 to the Emergency Departments of Study Hospitals from March 15-May 14, 2020**

| Characteristic | March | | | April | | | | May | | Overall |
| --- | --- | --- | --- | --- | --- | --- | --- | --- | --- | --- |
|  | 15-21 (n=564) | 22-28  (n=1550) | 29-Apr 4 (n=2173) | 5-11  (n=1277) | 12-18 (n=586) | 19-25 (n=310) | 26-May 2 (n=194) | 3-9 (n=166) | 10-14 (n=103) | n=6923 |
| Age, years | 58  (43-72) | 60  (44-72) | 63  (51-75) | 66  (54-77) | 68  (56-80) | 66  (52-78) | 68  (54-80) | 62  (43-73) | 59  (31-74) | 63  (50-76) |
| Female | 43.4% | 45.9% | 41.7% | 42.7% | 45.7% | 49.7% | 53.6% | 60.2% | 47.6% | 44.5% |
| Race/Ethnicity^1^ | | | | | | | | | | |
| White, non-Hispanic | 25.3% | 22.2% | 21.1% | 21.8% | 17.7% | 15.8% | 15.4% | 26.5% | 19.2% | 21.7% |
| Black, non-Hispanic | 30.5% | 31.5% | 30.9% | 28.4% | 20.8% | 36.8% | 12.8% | 11.8% | 10.6% | 29.3% |
| Hispanic | 42.1% | 43.2% | 45.0% | 46.9% | 61.5% | 47.4% | 71.8% | 60.3% | 70.2% | 46.4% |
| Comorbidities | | | | | | | | | | |
| Hypertension | 33.2% | 32.9% | 37.7% | 45.2% | 49.8% | 39.0% | 52.6% | 47.0% | 39.8% | 39.4% |
| Diabetes | 22.2% | 22.7% | 25.5% | 31.6% | 34.8% | 30.0% | 34.5% | 29.5% | 25.2% | 27.1% |
| Chronic pulmonary disease | 12.9% | 13.9% | 12.5% | 15.0% | 13.5% | 19.7% | 16.0% | 15.1% | 17.5% | 13.9% |
| Coronary artery disease | 8.7% | 8.5% | 10.8% | 15.3% | 16.6% | 11.9% | 16.5% | 13.3% | 13.6% | 11.7% |
| Obesity (BMI >30)^2^ | 42.1% | 41.4% | 36.6% | 32.0% | 29.9% | 26.1% | 28.7% | 32.4% | 26.7% | 35.2% |
| Days of symptoms prior to ED presentation^3^ | 4 (2-7) | 5 (3-7) | 7 (3-10) | 7 (3-10) | 7 (3-11) | 7 (3-14) | 7 (3-10) | 7 (2-14) | 3 (0-7) | 6 (3-9) |
| SARS-CoV-2 RT-PCR assay | | | | | | | | | | |
| cobas | 100.0% | 100.0% | 94.8% | 65.3% | 28.3% | 23.2% | 23.2% | 10.8% | 33.0% | 77.2% |
| Xpert Xpress | 0% | 0% | 5.2% | 34.7% | 71.7% | 76.8% | 76.8% | 89.2% | 67.0% | 22.8% |
| Admission to the hospital | 62.9% | 60.8% | 65.1% | 78.2% | 92.2% | 86.8% | 86.6% | 78.9% | 67.0% | 70.6% |
| COVID-19 therapies in hospitalized patients | | | | | | | | | | |
| Hydroxychloroquine | 63.1% | 68.2% | 72.1% | 75.4% | 58.0% | 37.2% | 27.4% | 10.7% | 1.5% | 63.7% |
| Remdesivir | 2.3% | 1.4% | 3.8% | 4.2% | 3.3% | 3.0% | 7.7% | 16.0% | 15.9% | 3.9% |
| Corticosteroids | 12.4% | 13.2% | 18.1% | 24.8% | 24.1% | 26.0% | 20.8% | 29.0% | 18.8% | 19.6% |
| IL-6 inhibitor | 1.7% | 2.6% | 4.9% | 6.4% | 6.9% | 7.1% | 9.5% | 1.5% | 0% | 4.9% |

Variables presented are % of total or median (interquartile range).

Abbreviations: Apr, April; BMI, body mass index; Mar, March; RT-PCR, reverse transcription-polymerase chain reaction.

^1^Race/ethnicity data were available for 2897 study participants (41.8% of total).

^2^Obesity data were available for 4884 study participants (70.5% of total).

^3^Duration of symptoms data were for 3497 study participants (50.5% of total).
