# Supplementary material for: Changes in SARS-CoV-2 viral load and mortality during the initial wave of the pandemic in New York City
Source: PLoS One. 2021 Nov 19;16(11):e0257979. doi: 10.1371/journal.pone.0257979 (PMC8604305; doi:10.1371/journal.pone.0257979)
Supplement: Attachment — Submitted filename: Response to Reviewers.doc [file pone.0257979.s005.doc]

**RESPONSE TO REVIEWERS**

**Journal Requirements**:

1) Please ensure that your manuscript meets PLOS ONE's style requirements, including those for file naming. The PLOS ONE style templates can be found at

<https://journals.plos.org/plosone/s/file?id=wjVg/PLOSOne_formatting_sample_main_body.pdf> and

[https://journals.plos.org/plosone/s/file?id=ba62/PLOSOne_formatting_sample_title_authors_affiliations.pdf](https://urldefense.proofpoint.com/v2/url?u=https-3A__journals.plos.org_plosone_s_file-3Fid-3Dba62_PLOSOne-5Fformatting-5Fsample-5Ftitle-5Fauthors-5Faffiliations.pdf&d=DwMGaQ&c=lb62iw4YL4RFalcE2hQUQealT9-RXrryqt9KZX2qu2s&r=654d60ikBk-o3I7mpMZQheIftwJLvrgDxmvRpXFRWlE&m=8aZvNCrwcAf6U3hzPxVpQS8htJE8QJdqiH_pPYme4D8&s=jE8FC0yonSiuHAZbGNV57UFg9Xx_UelT7sL9kT3akfI&e=)

Response: We have formatted the manuscript to meet PLOS ONE’s style requirements

2. Please include your full ethics statement in the ‘Methods’ section of your manuscript file. In your statement, please include the full name of the IRB or ethics committee who approved or waived your study, as well as whether or not you obtained informed written or verbal consent. If consent was waived for your study, please include this information in your statement as well.

Response: An ethics statement was added to the Methods section, including the IRBs who approved the study and that the study was conducted with a waiver of informed consent (lines 104-106, track changes version).

3. Please review your reference list to ensure that it is complete and correct. If you have cited papers that have been retracted, please include the rationale for doing so in the manuscript text, or remove these references and replace them with relevant current references. Any changes to the reference list should be mentioned in the rebuttal letter that accompanies your revised manuscript. If you need to cite a retracted article, indicate the article’s retracted status in the References list and also include a citation and full reference for the retraction notice.

Response: All references have been reviewed and edited for accuracy. We corrected the publication year for reference 18. No cited papers have been retracted.

**Reviewer #1:**

1) There are some typo errors and authors should use scientific words.

Response: We have reviewed the manuscript for typo errors and have ensured that scientific words were used throughout the manuscript.

2) If authors add some information related with other respiratory infections, value of this manuscript will be improved

Response: Unfortunately, we do not have data on other respiratory viruses that occurred at study hospitals during the study period. In fact, many of the hospitals did not test for other respiratory viruses during the initial wave of the pandemic to maintain the resources needed for the incredibly high testing volume for SARS-CoV-2.

**Reviewer #2**:

1) It would be great if authors could add one sentence about the reason of selecting those six hospitals in methods section.

Response: We added to a sentence in the Methods section that outlines why these hospitals were selected for the study (lines 92-96, track changes version).

2) In addition, authors could mention the exact start date of implementing non-pharmacological public health interventions to reduce interpersonal interactions and aerosol exposures in NYC. It could strengthen the argument in the discussion section.

Response: We added two sentences to the Discussion that outline the exact dates that non-pharmacological interventions were initiated in New York City (lines 303-305, track changes version).
